# Nomen est Omen: do antidepressants increase p11 or S100A10?

**DOI:** 10.1186/1747-5333-1-5

**Published:** 2006-04-13

**Authors:** Hari Manev, Radmila Manev

**Affiliations:** 1 The Psychiatric Institute, Department of Psychiatry, University of Illinois at Chicago. Chicago, Illinois 60612, USA

## Abstract

Occasionally, multiple names are given to the same gene/protein. When this happens, different names can be used in subsequent publications, for example in different research areas, sometimes with little or no awareness that the same entity known under a different name may have a major role in another field of science. Recent reports about the protein p11 presented findings that this protein, commonly known as S100A10, may play a crucial role in depression and antidepressant treatment mechanisms. One set of data showed an increased expression of this protein in the brain of mice treated with antidepressants. P11/S100A10 is only one of several S100 proteins expressed in the brain. Interestingly, it has been previously noted that antidepressant treatment increases the brain content of another S100 protein, S100B. It appears that up-regulating the brain content of various S100 proteins might be a common feature of antidepressants. In cells coexpressing S100A10 and S100B, these proteins may interact and exert opposite regulatory roles. Nevertheless, S100A10 is predominantly expressed in certain types of neurons whereas S100B is more abundant in glia. Thus, an interplay among multiple members of the S100 proteins might be important in determining the region and cell specificity of antidepressant mechanisms. Calling the p11 protein by its other name, S100A10, may prompt more investigators from different fields to participate in this new direction of neurobiological research.

## Introduction

What's in a name? ["*What's in a name? That which we call a rose by any other word would smell as sweet*." Act II, scene 2 from *Romeo and Juliet*; W. Shakespeare]. Sometimes more than it appears on the first glance. A choice of names, jargon, or terminology shapes the way we view the world [1]. Naming things is an integral part of scientific discovery. Newly discovered entities need names. The discoverers sometimes are indulgent in choosing names for their findings. Particularly playful is the *Drosophila *(fruit fly) research community when it comes to naming new genes. For example, *Cheapdate *is a gene that influences whether flies offered alcohol become quickly drunk, thus a "cheap date" [2]. *Indy *is an acronym for "I am not dead yet," based on a comical line in the movie *Monty Python and the Holy Grail*, and describes a gene that extends the life of flies [3].

These are examples of intentionally humorous and provocative names given to serious discoveries. More often, serious discoveries bear boring and technical names. Occasionally, multiple names are given to a same entity, exemplifying how sometimes the same discoveries can be made multiple times by groups unaware of each other's findings. When this happens, multiple names can be used in subsequent publications, for example in different research areas, sometimes with little or no awareness that the same entity known under a different name may have a major role in another field of science.

## "A new molecule to brighten the mood"

This title was used in a recent issue of *Science *[4] to popularly describe the scientific findings reported in the peer-reviewed portion of the same issue of this journal [5]. The published findings point to a novel interaction between a protein called p11 and serotonin (5-hydroxytryptamine; 5-HT) receptors called 5-HT_1B_. It was proposed that the reported protein-protein interactions might be involved in the pathobiology of depression (a psychiatric illness attributed in part to 5-HT disturbances) and in the mechanism of action of antidepressant drugs (many of which are selective 5-HT reuptake inhibitors). Various forms of the *Science*-generated catchy title about the "new molecule p11" have been adopted by other media outlets around the world and established their roots in the everlasting *www*, for example [6]. Is p11 indeed a completely new molecule when it comes to antidepressant effects?

## S100 proteins and antidepressants

The discovery by Svenningsson et al. [5] is important, and the route that led to the finding that 5-HT_1B _receptors directly interact with p11 and that p11 increases localization of 5-HT_1B _receptors at the cell surface has not been predicted based on previously published association between these proteins. Furthermore, the authors found that in the postmortem brain of depressed subjects p11 protein levels are lower compared to the samples from non-depressed subjects. Moreover, experimental animals treated with antidepressants had elevated brain levels of p11, suggesting that antidepressants could be helpful because they increase p11, which brings more 5-HT_1B _receptors to neuronal membranes to be available for activation by their neurotransmitter 5-HT. Is there any previous work that could link p11 to antidepressants? A PubMed search (January 20, 2006) on *"p11" & "antidepressant" *generated only 1 hit, the paper by Svenningsson et al. [5].

However, in their paper, Svenningsson et al. mentioned that p11 is also known by different names: "S100A10, calpactin I light chain, and annexin II light chain". It was proposed [4] that since p11 belongs to the family of S100 proteins and because these proteins can be regulated by glucocorticoids (stress hormones), the stress effects on p11 could explain the link between stress and the pathobiology of depression.

Whereas the PubMed searches on "*calpactin I*" & "*antidepressant*" and "*annexin II*" & "*antidepressant*" did not produce results, a search on "*S100*" & "*antidepressant*" resulted in 19 citations. The first (i.e., the oldest) report was from 1993 [7], pointing to a link between antidepressants, 5-HT_1A _receptors, and the glia release of S100B protein. Nevertheless, the concept of glia-secreted S100B as a mediator of antidepressant activity proposed in that paper was unrelated to the currently proposed association of antidepressants, 5-HT_1B _receptors, and the S100A10 protein.

The S100 protein family comprises 19 members [8], most of which are expressed in the brain [9]. The issue of systematically naming various S100 proteins has been recognized in the past and Schafer et al. [10] proposed specific and logical nomenclature of these proteins that has been widely accepted. According to this nomenclature, p11 is named S100A10. Interestingly, it was previously found that, similar to their stimulatory effect on the brain content of S100A10 [5], antidepressants also increase the brain, i.e., hippocampal content of S100B [11-13], and it has been proposed that S100B-related mechanisms could become targets for a novel antidepressant therapy [14,15].

In cells coexpressing S100A10 and S100B, these proteins may interact and exert opposite regulatory roles; e.g., on the assembly of the glial fibrillary acidic protein (GFAP) [8]. Since it appears that S100A10 is predominantly expressed in certain types of neurons, whereas S100B is more abundant in glia, S100 proteins might be important in determining the region and cell specificity of antidepressant mechanisms. Experiments designed to consider a more general role of S100 proteins in depression and antidepressant mechanisms as opposed to focusing solely on S100A10 might generate important new findings.

## Conclusion

It appears that up-regulating the brain content of various S100 proteins might be a common feature of antidepressants. The sophisticated set of experiments published by Svenningsson *et al*. suggests that p11/S100A10 may contribute to antidepressant activity by interacting with serotonin 5-HT_1B _receptors and by increasing their membrane presence and availability. In light of previously published studies it would be worth exploring whether S100B and other S100 proteins also affect a cell membrane insertion of some receptor proteins. The work by Svenningsson *et al*. [5] provides an important impetus for further clarifying the role of S100 proteins in depression, and calling their p11 protein by its more appropriate S100A10 name may prompt more investigators from different fields to participate in this exciting new research direction.
